# Mandatory vaccination of infants in France: Is that the way forward?

**DOI:** 10.1080/13814788.2018.1561849

**Published:** 2019-01-23

**Authors:** Henri Partouche, Serge Gilberg, Vincent Renard, Olivier Saint-Lary

**Affiliations:** a Conseil Scientifique du Collège National des Généralistes Enseignants (CNGE), Paris, France;; b Département de Médecine Générale, Faculté de Médecine, Sorbonne Paris Cité, Université Paris Descartes, Paris, France;; c Département de Médecine Générale, Faculté de Médecine, Université Paris-Est Créteil Val de Marne (UPEC), Créteil, France;; d Département de Médecine Générale, Faculté des Sciences de la Santé Simone Veil, Université Versailles Saint-Quentin en Yvelines (UVSQ), Montigny-le-Bretonneux, France;; e Centre de Recherche en Épidémiologie et de Santé des Populations, Université Paris-Sud, UVSQ, INSERM U1018, Université Paris-Saclay, Villejuif, France

**Keywords:** Mandatory vaccinations, vaccine hesitancy, immunization, trust

## Abstract

In this opinion paper, the authors argue that the extension of mandatory immunization of infants up to two years of age from three diseases (diphtheria, tetanus, poliomyelitis) to 11 diseases, introduced in France in January 2018, is not a sustainable response to the challenge of controlling vaccine-preventable diseases. In France in 2017, infant immunization coverage (IC) rates were sufficiently high or increasing (hepatitis B), except for measles, mumps and rubella (MMR) and meningococcus C disease. Even if vaccination obligation makes it possible to achieve the MMR IC objectives among infants, communication programmes and supported advice from GPs are essential for the catch-up of susceptible adults to obtain herd immunity. The impact of mandatory immunization on hesitancy remains uncertain, and it contradicts the evolution of the patient’s role in the governance of his own health and the principle of autonomy. Numerous studies have shown that interventions and advice from health professionals improve vaccine acceptance. To correct the poor implementation of some vaccination programmes by health professionals, strong communication and resources from health authorities are needed, rather than a retreat towards obligation. Reducing missed opportunities and increasing access to immunization are essential objectives. Finally, an immunization policy based on primary care and a patient-centred approach to each vaccination are more likely to reduce vaccine hesitancy, sustainably.

KEY MESSAGES

- Extended mandatory immunization of infants in France is not a sustainable response to the global challenges of controlling vaccine-preventable diseases.
- Large-scale communication and intervention programmes, directed towards the general public and professionals, are required to reduce vaccine hesitancy.
- Advice from GPs regarding each recommended vaccine, given through a patient-centred approach, has a positive impact on the decision to be vaccinated.

## Introduction

The extension of mandatory immunization of infants for 11 diseases, introduced in France in 2018 provides a ‘case study’ for public health. This opinion paper by members of the Scientific Council of the National College of Teachers in General Practice (CNGE; Box), reviews the factors that led the French health authorities to the use of the law. It provides a critical perspective on this change in immunization strategy for infants, on vaccine hesitancy in a context where a strong need for communication is expressed by patients and where the doctor–patient relationship is evolving towards shared decision-making.

## Brief historical background

Until the end of 2017, the French vaccination schedule combined mandatory and recommended vaccinations, which makes the distinction between obligation and recommendation difficult to understand for both health professionals and the general population. Diphtheria (D) and tetanus (TT) vaccinations were mandatory up to the age of 18 months and inactivated poliovirus vaccination (IPV) up to the age of 13 years. In Western Europe, most other countries had vaccination policies that were based solely on recommended vaccination programmes. Exceptions were Italy (with D, TT, IPV and hepatitis B (HepB) mandatory), Belgium (with IPV mandatory) and four of the 24 Swiss cantons (with D and IPV mandatory).

In May 2017, Italy extended its vaccination requirements to include the following 10 vaccines: D, TT, pertussis (acP), IPV, haemophilus influenzae type b infection (Hib), HepB, measles, mumps and rubella (MMR) and varicella (VAR) [1]. Subsequently, France opted for a simplification strategy by extending its requirements to include 11 infant vaccines. Since January 2018, all infants born in metropolitan France must have received obligatory D, TT, acP, IPV, Hib, HepB, MMR, pneumococcal disease (PCV) and meningococcus C disease (MenC) vaccines before the age of two years to enter a school, day-care, summer camp or other children’s community [2,3]. In the event of vaccine refusal, penal proceedings concerning the risk to one’s child and the exposure of others to preventable diseases may be initiated.

In 2017, a ‘citizen consultation’ was conducted by a committee of experts and user representatives, at the request of the Minister of Health. The mission was to determine how to respond to vaccine hesitancy that was causing low immunization coverage (IC). The authors of the report emphasized in their conclusion that strengthening public health policy in the field of vaccinations would eventually make it possible to lift the mandatory status of DD, TT and IPV and to base recommended vaccination on the understanding that it is in the best interest of all people, both individually and collectively. However, the authors ultimately recommended that vaccination requirements for children be temporarily extended (despite the opinion of the health professionals interviewed and half of the users) until a favourable perception of vaccinations could be achieved [4]. The studies focusing on the opinion of the French population and health professionals regarding mandatory immunization were sparse and did not make it possible to anticipate what attitudes would be if vaccination requirements were to end [5,6]. In the ‘2016 health barometer’ study of Gautier et al., 8.8% of parents said they would probably not vaccinate their child if the requirement were removed, and only 4% said they would certainly not [5]. Finally, the decision-making process of the Minister of Health was accelerated by an injunction of the Conseil d’État in February 2017. That injunction required that measures be taken within six months to provide vaccines with valences corresponding only to requirements (D, TT, IPV vaccine)—such vaccines were no longer manufactured at that time, and there were only vaccines combining recommended and mandatory valences—unless the law evolved by widening the scope of mandatory vaccinations [7].

In June 2017, the French National College of Teachers in General Practice (CNGE) stated its disagreement with this strategy, which does not respond to the global challenges of controlling vaccine-preventable diseases. In agreement with this position, an editorialist at the journal *Nature* noted that in its decision to mandate vaccination for many diseases, France provided a ‘case study’ for public health strategies in industrialized countries, as most of these countries have opted to make most vaccines recommended rather than mandatory [8]. In addition, a recent overview of mandatory immunization shows that there is limited evidence for the benefits of mandatory vaccination and that hard mandatory immunization may have unintended consequences [9].

## Will the obligation have an impact on vaccine hesitancy?

Individual decision-making regarding vaccination involves emotional, cultural, social, spiritual and political factors as much as cognitive factors [10]. Erroneous and misleading information from the media and social networks may have a negative impact on perceptions of vaccine risks and thus on decisions about vaccination [11]. Anti-vaccine leagues broadcast fake news that can affect a significant portion of the silent population by changing their subjective relationship to the social norm and by altering their confidence in health authorities [12,13]. In France, a series of health controversies occurred after the tainted blood scandal in the 1980s. The alleged link between hepatitis B vaccination and multiple sclerosis in the 1990s, the Mediator scandal, and the failure of the 2009 pandemic flu vaccination campaign have fuelled vaccine criticism and strengthened the influence of anti-vaccine leagues regarding controversies about adjuvants such as aluminium. With 41% of French individuals holding negative views of vaccines, vaccine safety is questioned in France more than in any other European country [14].

In this context, health authorities wanting to restore confidence in vaccines should have relied more on the lever of strong and reactive communication with the public and practitioners. By introducing an extended obligation, they have opted for a modification of the subjective standard (‘my child must have had his 11 vaccines because it is the law’), an action that also presupposes blind confidence among the public. This absolute measure raises ethical questions because it contradicts the evolution of the patient’s role in the governance of his own health; it also contradicts the principle of autonomy that is enshrined in the French law of 4 March 2002 on patients’ rights. Patients are demanding more information on the risk–benefit balance of each vaccination [15]. Moreover, the international literature highlights that the advice of health professionals and the guidelines of health authorities are the most-cited causes of vaccine acceptance [16]. Several studies have shown that general practitioners still have the confidence of patients and that their advice is still the leading factor in encouraging vaccination [17,18]. We know that it is through the doctor–patient relationship that trust in public health policies can be restored [10,16,19]. However, the health education work that health professionals have carried out over a long period will lose its effectiveness if health authorities decide to use the law to take back control of vaccination decision-making, even if the goal is to protect everybody. Furthermore, a socio-historical report has highlighted the propensity of mandatory vaccination to favour the emergence of anti-vaccine movements [20]. In an overview of mandatory immunization and based on the example of Serbia, MacDonald et al. [9] have shown that mandating vaccines may exacerbate negative vaccine sentiments and backfire for some who were hesitant prior to the law [9].

Furthermore, it should be added that this compulsory measure only concerns infants up to the age of two years. At the same time, communication regarding booster or catch-up vaccinations recommended for older children, adolescents and adults remain weak and underemphasized. Therefore, studies that evaluate vaccination requirements should focus not only on vaccination coverage but also on whether hesitation about recommended vaccines persists.

## Obligation is not the only way to improve immunization coverage

It is conceivable that in response to a major epidemic, targeted mandatory vaccination may contribute to a rapid increase in herd immunity. However, the extension of mandatory vaccination to 11 vaccines for infants in France has not been introduced as part of a public health emergency but rather as a response to ‘insufficient vaccine coverage, persistence of a preventable burden for some diseases and growing vaccine hesitancy in the French population’ [3].

Before the introduction of the obligation measure, the rate of primary IC for D, TT, and IPV among infants was 99% [21]. For some immunization programmes, immunization coverage was increasing before the obligation. This was the case with the HepB vaccine, for which IC increased from 47% in 2008 (the year the reimbursed hexavalent vaccine was introduced) to 90% in 2016 [21]. However, it was expected that two failed immunization programmes—MMR and MenC conjugate immunization—would increase their IC through mandatory immunization.

Regarding MMR, the objective of the WHO Global Strategic Plan to eliminate measles and rubella (postponed to 2020) requires that the IC be higher than 95%, with two doses required for children less than two years of age throughout each national territory. In France, the first dose is at 12 months and the second dose between 16 and 18 months. In 2015, the IC was 90.5% with one dose and 78.8% with two doses in children aged 24 months [21]. Except for Spain and Portugal, most Western European countries had also not achieved a two-dose vaccination coverage higher than 95% [22]. The measles epidemic in southwest France that began in November 2017 and extended to the first quarter of 2018 highlights the impact of a susceptible subject pool on epidemic dynamics. In this case, that pool was adults born after 1980 who did not receive two doses of MMR (because vaccination was introduced in 1983, the majority have never been in natural contact with the virus). Thus, even if the obligation makes it possible to achieve the objectives of MMR IC among infants, a more voluntarist policy of catching up among persons born after 1980 is also necessary. Additionally, the obligation does not resolve the paradox—identified by the French High Council on Public Health in 2013—of the coexistence of mandatory vaccines for infants and recommended vaccines for adults [23].

Concerning the MenC conjugate vaccination introduced in France in 2010, the French schedule was one dose for all subjects aged between one and 24 years. At the end of 2016, MenC IC data were 70.8% for two-year-olds, 35.7% for 10–14-year-olds, and 10.1% for 20–25-year-olds [21]. Among other European countries, vaccination schedules and catch-up strategies vary widely. Countries such as England, Ireland, Spain, and the Netherlands, which introduced the MenC vaccine in the early 2000s, have IC rates above 90% with an impact on case incidence [24]. French IC rates have not reached the level to guarantee the herd immunity that would protect children less than one year of age. In a cross-sectional observational study among GPs in 2014, Verger et al., showed that 51.7% of GPs recommended MenC conjugate vaccination to 12-month-old infants, and only 33.3% recommended the catch-up to 2–24 year-olds [25]. Nevertheless, most French general practitioners are in favour of vaccination, and they promote it as much as they are comfortable informing their patients of their position [26]. The MenC vaccine has not been communicated in a sufficiently focused and clear manner regarding its benefit/risk ratio at the individual and collective level [27]. The lack of ownership by GPs regarding the updated public health issues of invasive meningococcal infections may explain the slow pace at which this vaccination has been implemented among GPs. However, to our knowledge, no study focusing on this issue has been conducted to date. Thus, during a time of vaccine hesitancy, the use by health authorities of the lever of communication with health professionals and the population remains a priority.

Besides, other measures could have been taken to improve ICs. As in Germany and Austria where there is also is a tradition of generalized social insurance, in France health professionals initiate the organization of primary care. Infants and children are mainly cared for by GPs and paediatricians, which are few. Parents must pay the non-reimbursed portion of childhood vaccines at the pharmacy, except for those (the majority) who have supplementary health insurance. In the context of vaccine hesitancy, it is necessary to improve access to immunization by making vaccines free and available in health facilities (vaccines available in the refrigerators of medical practices) and to reduce missed opportunities to immunize during each contact with a primary healthcare professional.

## How to manage consultations in the era of mandatory vaccination for infants?

As shown by Yacub et al., ‘The most commonly cited reason for general population support for vaccination is healthcare professionals’ advice, although this category also includes the often false belief that vaccination is mandatory’ [16]. Vaccine obligation could have the advantage of a faster adherence to vaccination and would allow GPs to avoid listening to, informing and accompanying their patients in a patient-centred approach. Regarding the vaccination of infants, the decision would no longer be shared but rather imposed. Adhering to the law would close the interview (instead of opening it as in the motivational interview) and save consultation time. In a position paper, Colgrove wrote ‘laws serve as a critical safety net as well as a powerful symbolic statement of pro-immunization social norms’ and he added ‘both persuasion and coercion are necessary, and neither is sufficient’ [28]. However, since the work by Charles et al., the doctor–patient relationship has evolved towards a shared decision-making process and it is no longer possible to talk about persuasion today [29].

The French health authorities have indicated that the obligation is intended to be temporary and last for the period needed to restore the confidence of the general public [3]. However, with the expectation that the obligation will soon be lifted and to respect patients’ rights in a manner consistent with the other European countries that have opted for the recommendation, GPs will continue to advise their patients using a patient-centred, scientific, ethical, adult and responsible approach. It remains essential to respond to patients’ and parents’ concerns about vaccination through multiple strategies [30]. It has been shown that multifaceted interventions and presumptive attitudes are associated with higher vaccination rates [31,32]. In addition, a recent study has shown the effectiveness of motivational interview techniques during the postpartum stay on long-term vaccine coverage [33]. Finally, to assist GPs in communicating better with their patients, simple and clear recommendations from the health authorities are needed.

## Conclusion

The improvement of vaccination coverage can only be achieved if we give ourselves the real means to do so: large-scale communication and intervention programmes directed towards the public and professionals. The French National College of Teachers in General Practice is ready to participate in such efforts. It will be difficult, but it is certainly worth trying.

In this context, the vaccination obligation evokes H.L. Mencken’s aphorism: ‘Every complex problem has a solution which is simple, direct, plausible—and wrong’ [34,35].
